# Supplementary material for: Identification and Expression of Integrins during Testicular Fusion in Spodoptera litura
Source: Genes (Basel). 2023 Jul 15;14(7):1452. doi: 10.3390/genes14071452 (PMC10379305; doi:10.3390/genes14071452)
Supplement: Supplementary file 1 [file genes-14-01452-s001.zip › Figure S1.pdf]

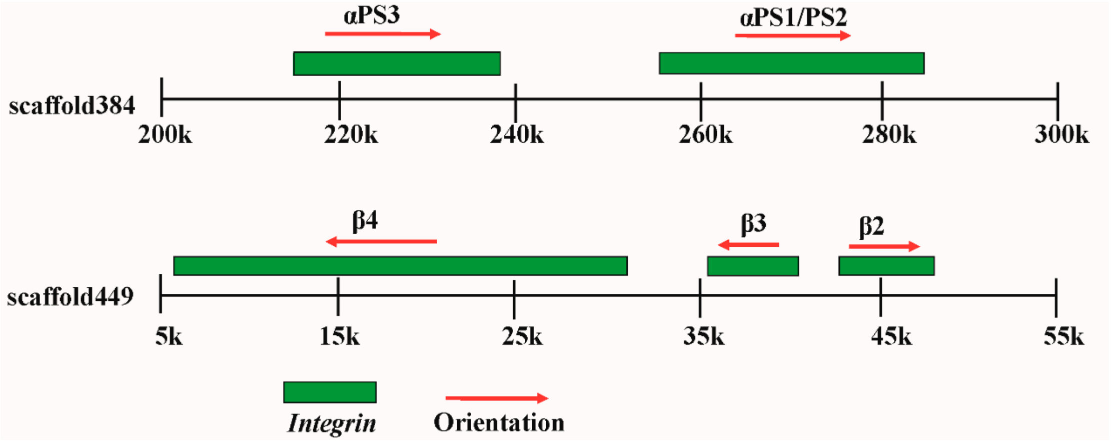

Figure S1 Locations of the integrins in the scaffolds of *S. litura* genome. The green boxes indicate the integrin localization and the red arrows indicate the transcriptional orientation.
